# Supplementary material for: A Likelihood Approach for Real-Time Calibration of Stochastic Compartmental Epidemic Models
Source: PLoS Comput Biol. 2017 Jan 17;13(1):e1005257. doi: 10.1371/journal.pcbi.1005257 (PMC5240920; doi:10.1371/journal.pcbi.1005257)
Supplement: S1 File — (TAR.GZ) [file pcbi.1005257.s014.tar.gz › HSPH_Online-SI-Revision/MSS10/n10-mild/n10-mild_NEW2_table-8.pdf]

1	newly 3 weeks spec	108.	173.52	{7., 435.}	112.352	0.566276	newly 3 weeks cumul	274.	421.25	{58., 922.}	93.9161	0.376527	newly 5 weeks spec	177.	210.45	{0., 519.}	92.2994	0.8215	newly 5 weeks cumul	569.	835.93	{58., 1906.}	94.9859	0.431596	newly 58 weeks cumul	3351.	1960.37	{58., 3622.}	52.1346	0.592804
2	newly 3 weeks spec	133.	391.14	{269., 485.}	194.09	0.45702	newly 3 weeks cumul	288.	709.59	{510., 925.}	146.385	0.381413	newly 5 weeks spec	307.	904.86	{712., 1077.}	194.743	0.463431	newly 5 weeks cumul	831.	2256.69	{1722., 2773.}	171.563	0.425957	newly 59 weeks cumul	4314.	8684.24	{7808., 9276.}	101.34	0.303057
3	newly 3 weeks spec	119.	450.73	{99., 858.}	291.336	0.532116	newly 3 weeks cumul	252.	867.49	{228., 1510.}	254.806	0.484995	newly 5 weeks spec	232.	670.7	{122., 1168.}	208.56	0.528654	newly 5 weeks cumul	651.	2148.68	{530., 3967.}	241.616	0.503634	newly 66 weeks cumul	4852.	4470.96	{2391., 5942.}	27.0387	0.183722
4	newly 3 weeks spec	134.	216.74	{64., 330.}	84.6716	0.379108	newly 3 weeks cumul	346.	474.18	{181., 733.}	57.6647	0.245536	newly 5 weeks spec	273.	386.64	{62., 575.}	76.4469	0.47485	newly 5 weeks cumul	805.	1156.22	{279., 1634.}	67.3814	0.298109	newly 61 weeks cumul	4621.	5518.81	{749., 8449.}	65.4995	0.399473
5	newly 3 weeks spec	76.	74.85	{41., 108.}	29.5395	0.141529	newly 3 weeks cumul	221.	216.02	{133., 295.}	23.6742	0.110407	newly 5 weeks spec	121.	72.46	{24., 118.}	43.5372	0.310324	newly 5 weeks cumul	442.	362.75	{203., 508.}	26.9434	0.148003	newly 41 weeks cumul	2815.	1497.8	{848., 2047.}	46.7922	0.309597
6	newly 3 weeks spec	89.	361.98	{20., 963.}	338.36	0.641921	newly 3 weeks cumul	165.	547.6	{76., 1295.}	257.285	0.508871	newly 5 weeks spec	215.	881.99	{31., 1957.}	342.879	0.771315	newly 5 weeks cumul	472.	2117.11	{173., 4978.}	353.068	0.643792	newly 67 weeks cumul	3503.	5519.8	{706., 8870.}	80.1376	0.413652
7	newly 3 weeks spec	131.	539.92	{404., 683.}	312.153	0.605716	newly 3 weeks cumul	306.	1042.22	{764., 1261.}	240.595	0.523942	newly 5 weeks spec	265.	993.16	{804., 1135.}	274.777	0.56922	newly 5 weeks cumul	790.	2824.92	{2148., 3332.}	257.585	0.547027	newly 63 weeks cumul	3676.	8736.86	{8216., 9221.}	137.673	0.375161
8	newly 3 weeks spec	120.	194.37	{42., 388.}	93.525	0.413016	newly 3 weeks cumul	269.	462.36	{140., 840.}	94.171	0.329456	newly 5 weeks spec	198.	260.29	{55., 494.}	73.803	0.378123	newly 5 weeks cumul	652.	955.47	{269., 1860.}	78.3635	0.291318	newly 59 weeks cumul	4528.	2705.64	{938., 3777.}	40.2902	0.317034
9	newly 3 weeks spec	122.	335.58	{215., 446.}	175.066	0.420774	newly 3 weeks cumul	305.	636.5	{409., 818.}	108.689	0.304155	newly 5 weeks spec	202.	744.05	{472., 975.}	268.342	0.552448	newly 5 weeks cumul	662.	1911.96	{1196., 2463.}	188.816	0.445778	newly 62 weeks cumul	4588.	8163.53	{7460., 8965.}	77.9322	0.249045
10	newly 3 weeks spec	146.	195.05	{51., 343.}	67.9795	0.327273	newly 3 weeks cumul	372.	543.15	{191., 935.}	70.3306	0.272504	newly 5 weeks spec	191.	197.61	{16., 420.}	63.8377	0.456617	newly 5 weeks cumul	713.	940.33	{298., 1567.}	63.4516	0.280234	newly 64 weeks cumul	3268.	1924.61	{403., 3264.}	42.2298	0.375407
11	newly 3 weeks spec	101.	1168.46	{320., 1597.}	1061.6	1.05316	newly 3 weeks cumul	225.	2663.66	{527., 4276.}	1088.08	1.00951	newly 5 weeks spec	200.	484.52	{149., 1043.}	155.53	0.406625	newly 5 weeks cumul	597.	4006.7	{1799., 4994.}	576.101	0.825511	newly 64 weeks cumul	2927.	4512.53	{3586., 5096.}	60.8866	0.250447
12	newly 3 weeks spec	49.	541.33	{144., 1224.}	1007.69	0.887112	newly 3 weeks cumul	71.	846.95	{239., 1836.}	1094.77	0.915719	newly 5 weeks spec	277.	1073.95	{516., 1703.}	291.289	0.585573	newly 5 weeks cumul	466.	2760.23	{1139., 5861.}	495.23	0.7187	newly 66 weeks cumul	3893.	8089.49	{6802., 9345.}	112.083	0.355461
13	newly 3 weeks spec	133.	101.61	{69., 132.}	27.9774	0.155111	newly 3 weeks cumul	290.	244.43	{171., 299.}	22.2103	0.111703	newly 5 weeks spec	295.	164.1	{104., 215.}	45.0373	0.295088	newly 5 weeks cumul	831.	540.41	{349., 675.}	36.3285	0.209273	newly 63 weeks cumul	4651.	6614.21	{5394., 7474.}	47.5728	0.189478
14	newly 3 weeks spec	178.	199.65	{158., 242.}	18.0955	0.0720414	newly 3 weeks cumul	407.	443.68	{368., 513.}	14.8845	0.061137	newly 5 weeks spec	248.	350.21	{271., 417.}	43.7863	0.155522	newly 5 weeks cumul	867.	1058.59	{857., 1252.}	25.1938	0.0970403	newly 59 weeks cumul	4541.	7228.16	{4918., 8192.}	61.1786	0.203674
15	newly 3 weeks spec	89.	235.16	{100., 330.}	175.753	0.47655	newly 3 weeks cumul	247.	490.05	{179., 704.}	111.81	0.349242	newly 5 weeks spec	149.	423.25	{187., 581.}	195.644	0.521351	newly 5 weeks cumul	522.	1245.55	{556., 1783.}	150.259	0.42625	newly 61 weeks cumul	4384.	5696.13	{3083., 8709.}	52.0244	0.235493
16	newly 3 weeks spec	118.	190.96	{24., 389.}	104.186	0.403601	newly 3 weeks cumul	242.	420.9	{82., 824.}	105.587	0.340058	newly 5 weeks spec	266.	277.19	{36., 542.}	68.6654	0.472661	newly 5 weeks cumul	698.	937.98	{171., 1802.}	81.1289	0.346252	newly 59 weeks cumul	4382.	2885.1	{392., 4058.}	36.2834	0.337947
17	newly 3 weeks spec	32.	34.89	{14., 59.}	44.6563	0.189333	newly 3 weeks cumul	75.	78.66	{39., 129.}	37.1733	0.161211	newly 5 weeks spec	45.	67.44	{22., 126.}	72.8889	0.241432	newly 5 weeks cumul	154.	195.29	{80., 333.}	51.3571	0.185286	newly 59 weeks cumul	3428.	6844.71	{4980., 7691.}	99.6707	0.293762
18	newly 3 weeks spec	96.	135.53	{9., 290.}	97.8229	0.54749	newly 3 weeks cumul	238.	339.38	{45., 635.}	85.916	0.354733	newly 5 weeks spec	159.	182.61	{0., 430.}	84.3711	0.690954	newly 5 weeks cumul	518.	691.51	{60., 1540.}	84.8398	0.3994	newly 59 weeks cumul	2823.	1886.74	{63., 3475.}	45.8569	0.475061
19	newly 3 weeks spec	250.	353.83	{44., 685.}	85.604	0.356767	newly 3 weeks cumul	538.	809.78	{136., 1499.}	88.7026	0.326018	newly 5 weeks spec	410.	363.97	{56., 603.}	41.8073	0.380801	newly 5 weeks cumul	1241.	1551.86	{301., 2673.}	65.6374	0.305586	newly 62 weeks cumul	4288.	2786.38	{810., 3749.}	35.1679	0.283441
20	newly 3 weeks spec	215.	179.95	{4., 395.}	60.6279	0.613009	newly 3 weeks cumul	419.	387.35	{54., 820.}	57.0048	0.377339	newly 5 weeks spec	303.	288.19	{0., 598.}	69.132	0.799845	newly 5 weeks cumul	946.	919.13	{54., 1875.}	62.0074	0.443219	newly 63 weeks cumul	4612.	2645.58	{54., 3994.}	44.144	0.53028
21	newly 3 weeks spec	171.	233.47	{19., 498.}	89.5731	0.488122	newly 3 weeks cumul	333.	476.98	{66., 985.}	88.9189	0.376861	newly 5 weeks spec	270.	376.66	{24., 733.}	94.4148	0.599658	newly 5 weeks cumul	859.	1167.99	{118., 2336.}	86.7066	0.422189	newly 63 weeks cumul	4235.	3642.51	{654., 8056.}	44.3998	0.375227
22	newly 3 weeks spec	162.	184.96	{80., 284.}	42.1605	0.210285	newly 3 weeks cumul	330.	417.74	{228., 613.}	43.4727	0.172865	newly 5 weeks spec	235.	281.01	{91., 410.}	49.0766	0.269999	newly 5 weeks cumul	771.	935.83	{436., 1383.}	43.7497	0.190391	newly 59 weeks cumul	3786.	5182.64	{1154., 7665.}	70.5325	0.313377
23	newly 3 weeks spec	145.	63.26	{1., 139.}	71.0345	0.866618	newly 3 weeks cumul	243.	133.61	{14., 286.}	63.4033	0.540975	newly 5 weeks spec	283.	130.26	{0., 279.}	78.1908	1.14266	newly 5 weeks cumul	754.	355.84	{16., 772.}	71.1857	0.708672	newly 64 weeks cumul	3569.	2638.16	{16., 5623.}	53.9047	0.709262
24	newly 3 weeks spec	208.	213.97	{144., 294.}	20.8029	0.0881396	newly 3 weeks cumul	535.	459.09	{336., 580.}	21.0262	0.104824	newly 5 weeks spec	227.	403.84	{290., 508.}	78.5903	0.238297	newly 5 weeks cumul	960.	1168.58	{843., 1496.}	28.0854	0.103524	newly 60 weeks cumul	2818.	7042.96	{5286., 8282.}	149.928	0.392262
25	newly 3 weeks spec	8.	114.33	{5., 288.}	1343.38	0.949701	newly 3 weeks cumul	23.	173.99	{14., 397.}	669.609	0.710165	newly 5 weeks spec	113.	499.82	{11., 1311.}	387.044	0.808929	newly 5 weeks cumul	209.	940.72	{41., 2466.}	388.44	0.652706	newly 69 weeks cumul	3078.	7316.47	{2861., 9304.}	155.069	0.562254
26	newly 3 weeks spec	173.	120.31	{5., 259.}	50.0636	0.554657	newly 3 weeks cumul	387.	387.82	{42., 786.}	47.46	0.326784	newly 5 weeks spec	234.	86.8	{0., 181.}	64.1197	0.864609	newly 5 weeks cumul	924.	579.64	{45., 1113.}	47.7576	0.403222	newly 58 weeks cumul	3097.	840.62	{52., 1528.}	72.857	0.756708
27	newly 3 weeks spec	144.	112.34	{0., 255.}	71.1806	0.702678	newly 3 weeks cumul	310.	284.13	{20., 597.}	62.4832	0.419473	newly 5 weeks spec	330.	118.3	{0., 242.}	74.703	1.1877	newly 5 weeks cumul	867.	525.37	{20., 1087.}	62.2157	0.566979	newly 61 weeks cumul	4074.	1309.66	{20., 4100.}	73.3965	0.883572
28	newly 3 weeks spec	96.	64.37	{25., 98.}	39.6563	0.260603	newly 3 weeks cumul	277.	199.12	{91., 289.}	33.3285	0.205323	newly 5 weeks spec	113.	54.65	{19., 95.}	52.5044	0.405527	newly 5 weeks cumul	500.	312.84	{139., 450.}	40.464	0.261906	newly 38 weeks cumul	2478.	797.99	{329., 1353.}	67.797	0.568313
29	newly 3 weeks spec	215.	292.36	{193., 383.}	40.9674	0.142562	newly 3 weeks cumul	511.	585.66	{394., 755.}	25.7573	0.101109	newly 5 weeks spec	312.	588.36	{357., 783.}	89.641	0.263074	newly 5 weeks cumul	1054.	1609.23	{1085., 2090.}	55.1736	0.18112	newly 61 weeks cumul	4420.	7667.27	{5445., 9059.}	74.4432	0.236613
30	newly 3 weeks spec	272.	287.86	{111., 426.}	39.7059	0.189934	newly 3 weeks cumul	607.	676.4	{261., 1056.}	38.659	0.168451	newly 5 weeks spec	287.	354.13	{135., 506.}	48.2822	0.210781	newly 5 weeks cumul	1151.	1363.07	{601., 2016.}	42.086	0.174986	newly 60 weeks cumul	4309.	3083.41	{1802., 3806.}	28.4426	0.175758
31	newly 3 weeks spec	58.	834.48	{187., 1652.}	1339.17	1.02739	newly 3 weeks cumul	93.	1195.22	{295., 2264.}	1185.4	0.982711	newly 5 weeks spec	156.	1694.96	{772., 2205.}	986.897	0.999631	newly 5 weeks cumul	351.	4381.57	{1624., 6859.}	1148.31	1.03672	newly 67 weeks cumul	3019.	8294.17	{6485., 9310.}	176.004	0.438276
32	newly 3 weeks spec	113.	129.91	{100., 163.}	21.5133	0.0845252	newly 3 weeks cumul	325.	378.72	{308., 453.}	18.9662	0.0737608	newly 5 weeks spec	137.	131.11	{91., 168.}	18.635	0.0864099	newly 5 weeks cumul	581.	640.27	{519., 770.}	15.6299	0.0630582	newly 40 weeks cumul	3452.	2053.67	{1658., 2411.}	40.5078	0.230609
33	newly 3 weeks spec	152.	124.9	{13., 253.}	53.2368	0.472386	newly 3 weeks cumul	297.	281.14	{44., 520.}	52.835	0.317026	newly 5 weeks spec	297.	215.08	{2., 448.}	51.697	0.621453	newly 5 weeks cumul	807.	665.8	{63., 1314.}	48.798	0.362907	newly 61 weeks cumul	4655.	3155.15	{148., 4556.}	43.6453	0.447692
34	newly 3 weeks spec	162.	301.17	{232., 385.}	87.0926	0.264656	newly 3 weeks cumul	346.	624.96	{479., 774.}	80.659	0.249479	newly 5 weeks spec	262.	577.54	{456., 692.}	121.969	0.342504	newly 5 weeks cumul	775.	1639.37	{1297., 1984.}	112.156	0.320327	newly 61 weeks cumul	4348.	7766.84	{7637., 7985.}	80.2686	0.260509
35	newly 3 weeks spec	234.	311.13	{122., 548.}	58.3889	0.360025	newly 3 weeks cumul	519.	681.56	{322., 1166.}	53.4721	0.253588	newly 5 weeks spec	339.	434.59	{115., 620.}	53.9617	0.396964	newly 5 weeks cumul	1155.	1500.14	{702., 2227.}	53.1654	0.285061	newly 63 weeks cumul	4450.	4478.96	{1130., 7781.}	50.88	0.358717
36	newly 3 weeks spec	156.	348.64	{83., 557.}	141.885	0.457722	newly 3 weeks cumul	432.	680.29	{201., 1037.}	77.2662	0.2793	newly 5 weeks spec	336.	678.55	{94., 1082.}	125.051	0.477891	newly 5 weeks cumul	978.	1882.97	{519., 2964.}	112.357	0.367585	newly 63 weeks cumul	4342.	6139.56	{2705., 9240.}	67.801	0.308457
37	newly 3 weeks spec	100.	274.48	{0., 542.}	227.84	0.770897	newly 3 weeks cumul	185.	552.87	{47., 1069.}	233.605	0.536818	newly 5 weeks spec	184.	332.07	{0., 716.}	149.408	0.944694	newly 5 weeks cumul	548.	1214.4	{47., 2408.}	174.23	0.603014	newly 66 weeks cumul	3080.	2335.78	{47., 4046.}	54.3623	0.630322
38	newly 3 weeks spec	166.	299.19	{208., 404.}	80.741	0.242438	newly 3 weeks cumul	393.	613.12	{431., 808.}	57.0331	0.184656	newly 5 weeks spec	256.	566.57	{389., 740.}	121.316	0.33261	newly 5 weeks cumul	843.	1605.68	{1102., 2127.}	90.4721	0.267005	newly 62 weeks cumul	4770.	8169.75	{7744., 8464.}	71.2736	0.233182
39	newly 3 weeks spec	121.	301.26	{17., 680.}	182.347	0.558797	newly 3 weeks cumul	299.	638.04	{49., 1334.}	145.398	0.423209	newly 5 weeks spec	257.	361.36	{1., 656.}	89.2218	0.636118	newly 5 weeks cumul	736.	1356.	{101., 2880.}	121.247	0.450743	newly 65 weeks cumul	3570.	2873.32	{123., 3988.}	28.949	0.356228
40	newly 3 weeks spec	88.	80.89	{36., 132.}	37.6705	0.215016	newly 3 weeks cumul	286.	248.31	{122., 375.}	30.3322	0.169209	newly 5 weeks spec	121.	74.48	{22., 135.}	45.3388	0.404448	newly 5 weeks cumul	519.	401.88	{189., 602.}	32.7514	0.204976	newly 36 weeks cumul	2280.	1059.97	{275., 1812.}	53.5101	0.443423
41	newly 3 weeks spec	60.	962.16	{162., 2010.}	1509.9	1.11001	newly 3 weeks cumul	169.	1485.84	{291., 3222.}	785.893	0.838688	newly 5 weeks spec	165.	1298.12	{482., 2010.}	694.305	0.935552	newly 5 weeks cumul	429.	4156.23	{1215., 7049.}	875.564	0.953581	newly 67 weeks cumul	3396.	6658.92	{3728., 8882.}	102.193	0.360957
42	newly 3 weeks spec	170.	127.23	{2., 271.}	56.7706	0.643314	newly 3 weeks cumul	363.	366.56	{48., 692.}	57.5207	0.358274	newly 5 weeks spec	211.	112.16	{0., 278.}	64.436	1.04735	newly 5 weeks cumul	769.	599.77	{53., 1205.}	52.277	0.431289	newly 59 weeks cumul	3469.	1125.78	{53., 3012.}	68.1908	0.801577
43	newly 3 weeks spec	65.	218.38	{0., 452.}	312.062	0.97933	newly 3 weeks cumul	145.	375.41	{12., 752.}	224.407	0.534261	newly 5 weeks spec	321.	218.81	{0., 653.}	84.3458	1.40076	newly 5 weeks cumul	595.	833.61	{12., 1976.}	134.795	0.732662	newly 65 weeks cumul	3665.	2064.32	{12., 4589.}	56.0229	0.915681
44	newly 3 weeks spec	156.	190.21	{24., 377.}	68.2115	0.410065	newly 3 weeks cumul	333.	432.66	{72., 800.}	66.3604	0.309345	newly 5 weeks spec	232.	263.37	{10., 505.}	71.4612	0.542811	newly 5 weeks cumul	768.	924.22	{124., 1749.}	63.9583	0.343822	newly 61 weeks cumul	4274.	2551.99	{152., 3822.}	40.7901	0.415626
45	newly 3 weeks spec	48.	85.19	{0., 99.}	206.854	1.0424	newly 3 weeks cumul	71.	127.69	{26., 163.}	200.465	0.794225	newly 5 weeks spec	143.	238.14	{0., 736.}	190.69	1.23073	newly 5 weeks cumul	285.	497.24	{3., 1221.}	196.625	0.978447	newly 68 weeks cumul	4420.	5950.91	{4., 9283.}	80.978	0.797046
46	newly 3 weeks spec	89.	667.37	{255., 996.}	652.528	0.818739	newly 3 weeks cumul	211.	1128.95	{462., 1658.}	439.	0.684813	newly 5 weeks spec	199.	1257.44	{656., 1647.}	533.859	0.773917	newly 5 weeks cumul	585.	3445.58	{1822., 4833.}	491.176	0.734603	newly 65 weeks cumul	3593.	7289.21	{5457., 9063.}	103.641	0.298975
47	newly 3 weeks spec	73.	228.48	{79., 420.}	222.466	0.503611	newly 3 weeks cumul	110.	394.7	{137., 712.}	264.873	0.515723	newly 5 weeks spec	151.	682.81	{211., 1197.}	360.298	0.690753	newly 5 weeks cumul	352.	1498.9	{441., 2642.}	332.75	0.608225	newly 65 weeks cumul	4475.	7406.26	{3917., 9140.}	75.8807	0.307119
48	newly 3 weeks spec	120.	104.53	{3., 205.}	58.125	0.536398	newly 3 weeks cumul	221.	231.3	{30., 428.}	61.2941	0.358172	newly 5 weeks spec	260.	192.56	{0., 447.}	63.3692	0.708199	newly 5 weeks cumul	652.	566.96	{38., 1258.}	59.1431	0.439721	newly 62 weeks cumul	3589.	2527.8	{38., 4073.}	38.5071	0.498191
49	newly 3 weeks spec	81.	142.23	{24., 254.}	108.951	0.424109	newly 3 weeks cumul	266.	430.23	{104., 776.}	88.688	0.30951	newly 5 weeks spec	116.	114.61	{3., 242.}	74.4397	0.604954	newly 5 weeks cumul	474.	672.76	{147., 1152.}	79.1899	0.324691	newly 65 weeks cumul	3702.	1229.86	{164., 2795.}	66.9573	0.677176
50	newly 3 weeks spec	137.	106.48	{4., 215.}	54.438	0.543543	newly 3 weeks cumul	367.	383.19	{45., 677.}	56.7003	0.334254	newly 5 weeks spec	235.	68.2	{0., 145.}	70.9787	1.0927	newly 5 weeks cumul	808.	533.69	{59., 979.}	45.9765	0.399619	newly 58 weeks cumul	2666.	689.94	{65., 1277.}	74.1208	0.781105
